# Daily Intake of Two or More Servings of Vegetables Is Associated with a Lower Prevalence of Metabolic Syndrome in Older People

**DOI:** 10.3390/nu16234101

**Published:** 2024-11-28

**Authors:** Gloria Cubas-Basterrechea, Iñaki Elío, Carolina González Antón, Pedro Muñoz Cacho

**Affiliations:** 1 Dietetic Section, Hospital Universitario “Marqués de Valdecilla”, 39008 Santander, Spain; 2 Research Group on Foods, Nutritional Biochemistry and Health, Universidad Europea del Atlántico, 39011 Santander, Spain; 3 Department of Health, Nutrition and Sport, Iberoamerican International University, Campeche 24560, Mexico; 4 NEXO Multidisciplinar Center, Department of Nutrition and Dietetic, 04004 Almeria, Spain; carolinaglan@gmail.com; 5 Teaching Department of Primary Care Management, Cantabrian Health Service, Instituto de Investigación Marqués de Valdecilla (IDIVAL), 39011 Santander, Spain; pedro.munoz@scsalud.es

**Keywords:** vegetables, metabolic syndrome, aged, Spain

## Abstract

Objectives: We sought to examine the correlation between the recommended consumption of at least two servings (400 g) of vegetables per day and the prevalence of metabolic syndrome (MetS) in an elderly population. Methods: This observational, cross-sectional, and descriptive study was conducted with 264 non-institutionalized people aged 65 to 79 years old. We adhered to the recommended guidelines for vegetable intake from the MEDAS-14 questionnaire, which has been validated for elderly populations at high cardiovascular risk. Diagnoses of MetS were made based on the criteria set forth by the International Diabetes Federation (IDF). Results: Among 264 individuals, who had a mean age of 71.9 (SD: 4.2) and comprised 39% men, the prevalence of MetS was 40.2%. A total of 17% of the participants adhered to the recommended vegetable consumption. Consuming the recommended amount of vegetables was correlated with a 19% reduction in the prevalence of MetS, to 24.4% from 43.4% among those with low vegetable consumption (*p* < 0.05). A main finding was that inadequate vegetable consumption was significantly associated with a higher prevalence of MetS (OR: 2.21; 95% CI: 1.06–4.63; *p* = 0.035), considering potential influences by nutritional (consumption of fruit and nuts) and socio-demographic (sex, age, and level of education) covariates. Conclusions: A beneficial inverse correlation was identified between the recommended vegetable intake and the prevalence of MetS. In contrast, inadequate vegetable consumption was revealed as an independent variable associated with the prevalence of MetS. Considering the very low adherence to the recommended vegetable intake we observed, encouraging increased vegetable consumption among older individuals, who have a high prevalence of MetS, is advisable.

## 1. Introduction

The global population is aging, with the proportion of individuals over 65 years of age having almost doubled, from 4.97% in 1960 to 9.54% in 2022, in the space of six decades [1]. The greatest percentage of people over 65 years of age (28.7%) is found in Japan, whereas the most aged populations in Europe are found in Italy (23.61%), Portugal (23.15%), and Finland (22.96%). Spain ranks 23rd in the world, with 20.3% of its population being over 65 years of age [1]. In Santander, Cantabria, in northern Spain, the percentage of people over 65 years of age in 2024 is also very high at 25.42% [2].

This high percentage of older people is associated with a high prevalence of metabolic risk factors involved in the development of metabolic syndrome (MetS) [3], which results in a high prevalence of MetS within this group [3,4,5]. MetS, as defined by the International Diabetes Federation (IDF) [6], is a set of five metabolic and inflammatory disorders: abdominal obesity, elevated triglycerides and blood glucose, increased blood pressure, and decreased high-density lipoprotein cholesterol (HDL-c). All of these conditions increase the risk of developing type 2 diabetes mellitus (DM2) and cardiovascular disease (CVD) in adults. A prerequisite for the diagnosis of MetS is abdominal obesity and at least two of the other four risk factors mentioned [6]. The pathogenesis of MetS is associated with genetic and acquired factors that trigger oxidative stress, cellular dysfunction, and systematic inflammation [7].

Modifiable factors such as dietary habits are key to the prevention and progression of MetS. Vegetables are widely designated as “protective foods” within healthy dietary patterns, such as the Mediterranean Diet (MedDiet), which has been confirmed to be one of the main strategies for the prevention and treatment of MetS [8,9]. The importance of vegetables in this way can be attributed to their richness in vitamins (vitamin C), minerals (potassium), essential fatty acids, amino acids, and bioactive components, such as phytochemicals (polyphenols) and fiber [10]. The low caloric value of vegetables, together with their high fiber and polyphenol contents, aids in maintaining a healthy body weight and the reduction in abdominal obesity [10,11]. Phytochemicals in particular confer health benefits due to their anti-inflammatory and antioxidant effects [12]. In consuming the recommended amount of vegetables in terms of the number of servings consumed and the frequency of their consumption, these dietary components have been associated with the prevention and treatment of chronic inflammation-related and MetS-associated pathologies [13,14,15]. In this sense, polyphenols enhance insulin sensitivity, regulating blood sugar levels [14]; polyphenols also lower blood pressure thanks to their potassium content [16] and prevent dyslipidemia due to their high fiber composition [17].

Instead, low vegetable consumption has been associated with the prevalence of three or more risk factors for MetS (abdominal obesity, arterial hypertension (AHT), hyperglycemia, and low HDL-c levels) [18]; consequently, low vegetable consumption is associated with an increased risk of MetS and CVD.

Therefore, an inadequate vegetable intake could constitute a modifiable factor for the presence of MetS in the large group of the older population [19,20], for whom vegetable consumption tends to be low [21,22,23,24] and MetS prevalence tends to be high. Measures to increase vegetable consumption among the elderly are justified on this basis to enable healthy aging [25].

The aim of this study was to examine the relationship between adherence to the recommended intake of at least two servings (400 g) of vegetables per day and the prevalence of MetS in an elderly population.

## 2. Materials and Methods

### 2.1. Study Design

This study employed observational, cross-sectional, and descriptive methods.

### 2.2. Participants

The study population comprised non-institutionalized elderly people aged 65 to 79 years old and living in Santander (Cantabria). In January 2023, the population within this age bracket totaled 31,334 individuals [26]. The Granmo v.7.10 software for finite populations was utilized to compute the sample size [27]. At an alpha risk of 5% and a beta risk of 20% with bilateral contrast, 73 individuals who consumed the recommended amount of vegetables (≥2 servings/day) and 146 individuals who consumed <2 servings of vegetables/day were assessed in order to identify a minimum difference of 20% between patients with and without MetS. As indicated by previous studies, one of these groups was expected to reach a proportion of 40% [20]. The ARCOSENE approach was employed.

The study population consisted of patient groups from four physicians affiliated with three Primary Health Care Centers (PCCs) in Santander (Cantabria) under the Cantabrian Health Service (CHS). To acquire the sample, a three-layer sampling method was employed: First, the three PCCs in Santander with the highest populations of patients aged 65 and older were identified. Subsequently, a purposive medical quota was sampled from each of the three designed centers; specifically, the opportunity to participate was offered to the coordinator of each of these PCCs, and from the center with the greatest number of patients within this age group, a second medical quota recommended by the coordinator of the PCC was chosen. Finally, a random sample of individuals, stratified by sex and age (65–79 years old), was selected using systematic sampling.

Following random and systematic sampling, this study commenced with 556 individuals; however, 292 were unable to participate for various reasons. Of these, 106 were excluded by physicians due to the applicability of the exclusion criteria, primarily mental and cognitive decline (>4 errors in the Pfeiffer test [28]) and acute issues with maintaining an upright posture. Letters were sent to the home addresses of the individuals selected via the CHS, informing them about this study, followed by telephone contact. However, 68 individuals were unreachable, and 65 declined to participate in this study. Ultimately, 53 people were excluded due to their CHS health cards lacking biochemical blood and/or prescription data from the past twelve months, as these data were pertinent to the diagnostic variables required to ascertain the prevalence of MetS according to the IDF criteria [6].

Consequently, after the application of the selection criteria, a final sample of 264 participants remained (men: 39%; women: 61%).

### 2.3. Socio-Demographic Characteristics

The patients’ socio-demographic characteristics were examined, including sex, age group, marital status, and level of education. Three age groups were established: 65–69 years old, 70–74 years old, and 75–79 years old. Marital status comprised four categories: married/partnered, separated, widowed, and single. Their educational levels were classified as university, secondary school, primary school, or incomplete primary school.

### 2.4. Diagnosis of Metabolic Syndrome

The IDF criteria were employed for the diagnosis of MetS, which stipulate that abdominal obesity is a prerequisite. Waist circumference was measured in the midpoint between the lower costal border and the top edge of the iliac crest in the standing position [29]. Given the challenges in taking this measurement in elderly individuals, particularly those who are overweight or obese, an anatomical reference point was established 2.5 cm above the umbilicus. This reference point facilitated measurement and was previously identified as the most accurate indicator of abdominal adipose tissue in older people [30]. Abdominal obesity in European individuals is defined as a waist circumference of ≥94 cm in men and ≥80 cm in women. Furthermore, a diagnosis of MetS requires two or more of the following parameters to be present: arterial hypertension (AHT) (130/85 mmHg, either treated with medication or diagnosed); fasting hyperglycemia (≥100 mg/dL or a prior diagnosis of DM2 or treatment); hypertriglyceridemia (≥150 mg/dL or under treatment); and low HDL-c (<40 mg/dL in men and <50 mg/dL in women or under treatment).

### 2.5. Instruments

#### 2.5.1. Adherence to Recommended Vegetable Consumption

The MEDAS-14 questionnaire, which has been validated for individuals aged 55–80 years old at elevated cardiovascular risk, is a fast tool to assess adherence to the MedDiet [31]. The brief screener containing 14 questions has a positive correlation with the frequency of consumption of items deemed healthy, such as vegetables, while demonstrating an inverse relationship with the intake of unhealthy foods, identifying items for improvement individually. Adherence to the MedDiet is deemed “good” if a test score is ≥9 points and “low” if it is ≤8 points [32]. We used the vegetable-related item from the MEDAS-14 questionnaire, which recommends the consumption of two or more servings of vegetables daily, with at least one of these servings including salad or raw vegetables. One serving constituted 200 g here, whereas side dishes and half servings were considered half portions. This recommendation for vegetable consumption aligns with that suggested by the Spanish Society of Community Nutrition (SENC) for older people, which is 2–3 servings per day, with each serving comprising 150–250 g [33]. To ascertain the participants’ consumption of vegetables and enhance their understanding of portion sizes, the dietitians–nutritionists (interviewers) utilized images showing portions of 200 g (one portion) and 100 g (a half portion) of various vegetables, except for potatoes. The study population was categorized into two groups: individuals who consumed vegetables in the amount recommended (≥2 servings per day) and those who had an inadequate consumption of vegetables below the recommended level (<2 servings per day).

#### 2.5.2. Assessment of Diagnostic Parameters for Metabolic Syndrome According to the IDF Criteria

A SECA^®^ model 203 (SECA, Hamburg, Germany) ergonomic tape with millimetric precision was utilized to measure the participants’ waist circumference, based on which diagnoses of abdominal obesity were made based on a comparison of the results with the reference values established for the European population by gender [6]. Hypertension was determined by measuring patients’ blood pressure using the OMRON M3^®^ Comfort automatic arm blood pressure monitor (Omron, Shimogyo-ku, Kyoto, Japan) in accordance with a strict protocol and by comparing the results with established diagnostic values [6]; additionally, the inclusion of one or more type of antihypertensive medication on a patient’s CHS health card facilitated diagnosis. Diagnoses of hyperglycemia were made utilizing fasting blood glucose data (and comparing them to the diagnostic values) and/or mentions of medication being prescribed for DM2 in the CHS health cards. The results on triglyceride levels in the blood and how they compared with the reference values [6] and/or triglyceride medications being listed on a patient’s CHS health card were used to determine whether an individual had hypertriglyceridemia. Low HDL-c levels were detected using HDL-c blood data from patients’ CHS health cards and comparing them to the diagnostic value established by the IDF criterion [6].

### 2.6. Statistical Analysis

Quality variables were characterized by frequencies and percentages, whilst quantitative variables were represented by the arithmetic mean and standard deviation in the descriptive analysis. To establish differences between qualitative variables, the chi-square test was used. The odds ratio was employed to measure the association between the independent variables and the dependent variable MetS, while statistical significance was assessed using the Wald test.

We employed logistic regression analysis to determine the variables associated with MetS. The recommended intake frequencies in the MEDAS-14 questionnaire were used as reference categories, as indicated in Table S1. Additionally, socio-demographic variables such as sex (reference women), age (years), and education level (reference university) were included. Variables with *p* < 0.25 in univariate analysis were incorporated into the multivariate analysis, adhering to the method established by Hosmer and Lemeshow [34] and applied by other authors [35]. The main independent variable was vegetable consumption, and the control variables were the consumption of fruits and nuts, which had shown an association with MetS in a previous study conducted in this same population, using the MEDAS-14 questionnaire [36]. Also, essential socio-demographic variables such as sex, age, and education level were included. To select the final model, the automatic variable selection procedure was used with the backward method (model M1). To compare the predictive capacity of the different models, the area under the ROC curve was used. It is difficult to label a certain range of ROC curve area magnitudes as “poor” and “good” because it depends on the disorder and clinical application [37]. In this case, it may be appropriate to consider an AUC greater than 0.70–0.75 as desirable.

For the analyses of the above data, we used SPSS 25 (IBM Co. Released 2017. IBM SPSS Statistics for Windows, Version 25.0, Armonk, NY, USA: IBM Corp) and MedCalc^®^ Software version 22.023 (MedCalc Software Ltd., Ostend, Belgium; https://www.medcalc.org; accessed on 29 October 2024).

## 3. Results

### 3.1. Socio-Demographic Characteristics

Table 1 shows the socio-demographic characteristics of the population investigated in terms of gender, age group, marital status, and level of education.

### 3.2. Adherence to Recommended Vegetable Consumption

In the older group studied (*n* = 264), the level of adherence to the recommended intake of two or more servings of vegetables per day was 17%. According to gender, the percentage of women who consumed the recommended amount of vegetables (21.1%) was significantly higher than the percentage of men who did (10.7%) (*p* < 0.05) (Table 2). There were no other significant differences according to the socio-demographic variables (age group, marital status, and level of education) or MetS diagnostic variables (abdominal obesity, MetS diagnostic parameters, and number of MetS diagnostic variables).

### 3.3. Prevalence of Abdominal Obesity

The mean waist circumference by gender was compared with the diagnostic values of abdominal obesity by gender. The average waist circumference in men (*n* = 103) was 102.6 (±SD: 10.9), whereas in women (*n* = 161) it was 90.7 (±SD: 13.6) (*p* < 0.001). In both cases, the mean waist circumferences exceeded the diagnostic thresholds for abdominal obesity (≥94 cm in men and ≥ 80 cm in women). The prevalence of abdominal obesity observed was 78.8%, without a significant difference by gender at 77.7% in men and 79.5% in women (Table 3). There were no significant differences by age group.

### 3.4. Prevalence of Metabolic Syndrome

The prevalence of MetS was 40.2% (Table 3), with a significantly higher rate in men (*n* = 103) at 47.6% compared to women (*n* = 161) at 35.4%, (*p* < 0.05). There were no statistically significant differences in the prevalence of MetS by age group.

In terms of the prevalence of diagnostic variables related to MetS, AHT was the most prevalent at 79.5%. The second highest prevalence was found for hyperglycemia at 31.8%. The prevalence of the remaining diagnostic variables for MetS was considerably lower, with a prevalence of 23.4% recorded for hypertriglyceridemia and a prevalence of low HDL-c of 19.7% (Table 3).

Concerning the number of diagnostic variables for MetS excluding abdominal obesity, it was observed that for 14.4% of the participants, none of the other diagnostic variables applied, and one other variable applied to 38.6% of them. Subsequently, two diagnostic variables for MetS applied to 27.6% of the participants; three variables applied to 15.2% of them; and the percentage of individuals to whom four diagnostic parameters applied was the lowest at 4.2% (Table 3).

### 3.5. Association Between Vegetable Consumption and the Prevalence of Metabolic Syndrome

**Figure 1:**
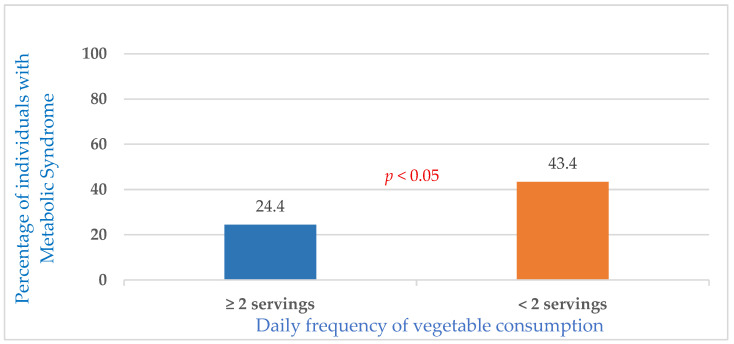
Association between the frequency of vegetable consumption and the prevalence of metabolic syndrome.

Consuming two or more servings of vegetables per day, in line with the recommendations, was associated with a prevalence of MetS of 24.4%, whereas consuming fewer than two servings per day was associated with a 19% higher prevalence of MetS (43.4%), with a significant difference (*p* < 0.05) (Figure 1).

As shown in Table 4, in the univariate logistic regression analysis, of the 14 nutritional variables from the MEDAS-14 questionnaire, only three were found to be significant (*p* < 0.25): consumption of vegetables in the amount of <2 servings/day (OR: 2.37; 95%CI: 1.14–4.92; *p* = 0.021); consumption of fruit in the amount of <3 pieces/day (OR: 1.35; 95%CI: 0.82–2.23; *p* = 0.237); and consumption of nuts in the amount of <3 portions/week (OR: 2.04; 95% CI: 1.21–3.42; *p* = 0.007).

From the socio-demographic variables analyzed (sex, age, and level of education), only the sex (reference women) was significant: the men had a 66% higher prevalence of MetS than women (OR: 1.66; 95% CI: 1.00–2.74; *p* = 0.049).

For a multivariate regression logistic analysis, in the M0 model, the variables that had been significant in the univariate logistic regression analysis (vegetables, fruit, and nuts) and all the essential socio-demographic variables were included.

As shown in Table 5, when controlling for the previous variables, inadequate vegetable consumption was related to a higher presence of MetS on the edge of meaning (OR: 1.96; 95% CI: 0.98–4.17; *p* = 0.082). The consumption of nuts below the recommendation was associated with a higher prevalence of MetS (OR: 1.93; 95% CI: 1.13–3.28: *p* = 0.016).

After applying the procedure of automatic variable selection using the backward method (model M1), only two variables were retained (low consumption of vegetables and nuts). Both variables were identified as independent contributors to MetS prevalence in this population: inadequate consumption of vegetables (OR: 2.21; 95%CI: 1.06–4.63; *p* = 0.035) and inadequate consumption of nuts (OR: 1.95; 95% CI: 1.15–3.29; *p* = 0.013).

The area under the ROC curve (AUC) values in the M0: 0.641 and M1: 0.61 were quite similar.

## 4. Discussion

### 4.1. Adherence to the Recommended Vegetable Consumption

The level of vegetable consumption in our study population was low, with merely 17% of the participants complying with the guideline on consuming at least two servings of vegetables per day. However, this low level of vegetable consumption aligns with previous findings on the dietary patterns of the population of Cantabria, as this region of Spain ranks third lowest in its vegetable consumption (at 48.10 kg/person/year) [38]. This diminished vegetable intake also aligns closely with the results of the PREDIMED-Plus study [39], which showed reduced vegetable consumption among the population of northern Spain.

The low consumption of vegetables in older people can be attributed to physiological factors such as loss of appetite, oral health problems, and tooth loss, as well as complications related to polypharmacy, mobility problems affecting shopping habits, and living alone [24].

The prevalence of adequate vegetable consumption was markedly low for both genders, although it was significantly higher in the women (21.1%) compared to the men (10.7%) at *p* < 0.05 (Table 2), as is shown in previous studies [21,23].

### 4.2. Prevalence of Metabolic Syndrome

The prevalence of MetS among the participants according to the IDF criteria was 40.2 [6], with similar data found for the population of Spanish people over 65 years of age enrolled in the ENRICA study (42.3%) [5]. Meanwhile, these data differ from those obtained in other parts of the world using different diagnostic criteria, such as Mexico (72.9%) [40], Brazil (66.1%) [41], Iran (51.7%) [42], and the United States (48.6%) [43].

MetS is not a disease but it is a chronic syndrome with a clustering of individual metabolic risk factors including abdominal obesity, hyperglycemia, hypertriglyceridemia, hypertension, and low high-density lipoprotein cholesterol levels that could increase the prevalence of DM2 and CVD [44]. For the diagnosis of MetS by the IDF criteria, abdominal obesity is indispensable as well as at least two of the four risk factors [6]. Therefore, the high prevalence of MetS obtained in this study is related to the fact that 78% of the people had abdominal obesity and 47% had two (27.6%), three (15.2%), or four (4.2%) diagnostic variables for MetS. The most prevalent diagnostic variables for MetS were AHT (79.5%) and hyperglycemia (31.8%) (Table 3). By sex, the prevalence of MetS in men was significantly higher (47.6%) than in women (35.4%) (*p* < 0.05). MetS is characterized by an increase in oxidative stress, which is associated with impaired inflammation, vascular disfunction, atherosclerosis, and a deregulation of the innate immune system [45]. It has been shown previously in several studies that AHT and hyperglycemia values allowed a better prediction of increased CVD risk than MetS itself in older people, due to the important role of insulin resistance in promoting chronic inflammation and atherosclerosis [46,47,48,49]. Also, a retrospective study in a large sample of the Korean population developed by Sung et al. showed a linear association between the number of diagnostic variables for MetS and risk of cardiovascular mortality, ranging from 1.99 for one variable to 2.98 for four–five variables [49].

### 4.3. Association Between Vegetable Consumption and the Prevalence of Metabolic Syndrome

An inverse association was found between vegetable consumption and the prevalence of MetS. This study revealed that the prevalence of MetS among individuals who consumed fewer than two servings of vegetables per day was 43.4% and was 19% higher than the prevalence of MetS among those who adhered to the recommended level (24.4%) (*p* < 0.05%) (Figure 1). The most relevant finding was that inadequate consumption of vegetables acquires its own significance in being associated with a higher prevalence of MetS, which was equivalent to a 2.21 times higher risk of prevalence of MetS (OR: 2.21; 95%CI: 1.06–4.63; *p* = 0.035). This held even after controlling for the potential confounding influence of socio-demographic variables (sex, age, and level of education) and the dietary consumption of other relevant foods, such as fruit and nuts, analyzed by the MEDAS-14 questionnaire in a previous study in the same population [36], with low nut consumption identified as another independent variable for the presence of MetS (Table 5). Based on the area under the ROC curve (AUC) values, although it is not the objective of this study, the predictive capacity was not satisfactory in either of the two models (M0: 0.641 and M1: 0.61). Both models had low predictive capacity (<0.70) despite the fact that M1 had a smaller number of variables, which suggests that there are more relevant variables that have not been included, such as physical exercise, socioeconomic status, and total calorie intake. The recent study by Papaioannou et al. [20] revealed analogous findings in older people (65–70 years old), indicating that low vegetable consumption significantly increased the likelihood of MetS (OR: 1.47; 95% CI: 1.04–2.07). In addition, inadequate vegetable consumption takes on its own importance as the independent variable for the prevalence of MetS, independent of physical activity or a sedentary lifestyle in older people [20]. Both studies were conducted on older people and confirmed that inadequate vegetable consumption was significantly associated with a higher prevalence of MetS in this group of people. Even in those with comorbidities, the risk of MetS proved to be significantly lower in individuals with a high intake of white and red vegetables (OR: 0.77; 95% CI: 0.57–0.91) compared to those with a low intake of these types of vegetables [13].

Oxidative stress and inflammation are interconnected conditions that characterize the pathophysiology of MetS and diseases associated with it [45,50]. It is well known that the consumption of vegetables—which are characterized by a nutritional composition high in vitamins (mainly A, B, and C) and minerals (selenium and potassium), rich in fiber, and low in fat [51,52,53], along with bioactive phytochemical contents with antioxidant and anti-inflammatory properties (such as polyphenols)—influences the diagnostic parameters for MetS positively and thereby reduces MetS risk [15,54]. Polyphenols are secondary metabolites synthesized by plants that potentially influence metabolic processes [54], and the 8000 that have been identified thus far are divided into flavonoids (flavones, flavonols, isoflavones, flavanones, flavanols, and anthocyanins) and non-flavonoids (phenolic acids, stilbenes, and lignans) [54]. Meanwhile, vegetables are one of our main sources of vitamin C, the antioxidative and anti-inflammatory properties of which are associated with mechanisms of action that can reverse MetS [50].

The following paragraphs describe how adequate vegetable consumption affects the various metabolic risk factors of MetS.

The elevated prevalence of abdominal obesity identified in the population studied (78.8%) (Table 3) may facilitate adipose tissue dysfunction (due to hyperplasia and hypertrophy of the adipose tissue with the release of inflammatory mediators), insulin resistance, the development of metabolic comorbidities and a higher prevalence of MetS [55]. On the other hand, consuming vegetables is associated with a reduction in abdominal obesity due to their high fiber content, which increases intestinal volume and encourages slower food consumption, thereby increasing satiety and reducing caloric intake. This results in diminished absorption of metabolizable energy in the intestines and improves the intestinal microbiota, facilitating a slimmer phenotype [39]. The high antioxidant/anti-inflammatory content in vegetables may help to reduce the low-grade inflammation associated with visceral adiposity [56]. In addition, greater adherence to adequate vegetable consumption in older people had a confirmed association with a lower BMI and waist circumference after 7.1 years of follow-up in previous research [57]. In this sense, the high flavonoid content of vegetables, like that found, for example, in green leafy vegetables, broccoli, tomatoes, celery, and parsley, may contribute to weight maintenance [11].

Polyphenols are known for being anti-diabetic bioactive compounds [58] and boosting insulin sensitivity, which improves insulin resistance by reducing postprandial glycemia, modulating glucose transport, protecting pancreatic β-cells from damage, and affecting insulin signaling pathways [59]. The latter mechanism is particularly significant because chronic activation of pro-inflammatory pathways in target cells responsible for the action of insulin can bring about obesity, insulin resistance, and DM2 [60]. Moreover, the polyphenols in vegetables can improve frequent diabetic complications, such as vascular dysfunction and coronary diseases [61].

A recent meta-analysis confirmed that vegetable consumption was associated with a reduction in both systolic and diastolic blood pressure [62]. This beneficial effect on blood pressure has been linked to the high fiber content and bioactive components of vegetables; high flavonoid intake in particular has been connected to improved endothelial vasodilation [63] and reduced inflammation and oxidative stress [19,64].

Vegetables are abundant in components that prevent dyslipidemia, such as fiber, phytosterols, antioxidants, and polyphenols [65]. Given its viscosity, fiber content has been identified as a factor that can lower triglyceride levels [17] in slowing gastric emptying, disrupting fat emulsification and micelle formation in the gastrointestinal tract and thus reducing the availability of bile acids in circulation. In addition, soluble fiber can be fermented by the gut microbiota to produce metabolites such as short-chain fatty acids, which regulate genes related to lipid metabolism and the postprandial triglyceride response [17]. Our study examined tomato consumption, characterized by its richness in lycopene, a well-studied terpene-derived carotenoid, which was shown to have a positive association with HDL-cholesterol levels [66,67]. Due to its antioxidant/anti-inflammatory activity, lycopene was found to contribute to beneficial changes in the components of MetS [67,68].

The multi-morbidity that characterizes MetS leads to each of those diseases being aggravated (synergism) and concomitantly to increased mortality [69]. MetS and its associated complications, such as CVD, are also characterized by chronic low-grade inflammation [70]. Therefore, the consumption of vegetables at the recommended level may have an important role in ameliorating MetS-associated pathologies, although the exact mechanisms behind this are still unknown [19]. In this vein, a recent prospective study of 48,632 individuals followed for 14 years found that increased vegetable consumption was associated with a reduction between 11 and 12% in cardiovascular mortality risk [71].

An inverse relationship between vegetable consumption and inflammatory biomarkers (IL-6) has been identified in older people [19,25,65]. The rich antioxidant contents in vegetables are able to alleviate the excess production of free radicals caused by normal-aging-related oxidative stress [72], potentially promoting healthier aging and lowering age-related systemic inflammation [19,25].

Therefore, given that adequate vegetable consumption has been associated with a significant reduction in the risk of MetS [73], health strategies that encourage eating the recommended quantity of vegetables and an increased variety of types of vegetables must be implemented [74]; moreover, it is important to take the wide range of constituents into account when discussing the positive impact of vegetable consumption on MetS [75]. Also, how the method of preparation affects vegetables that are consumed (e.g., whether they are raw or cooked) should also be considered [22].

Given that this was an observational study, inherent limitations apply, and cause–effect relationships could not be determined as in prospective studies. For this reason, the participants’ dietary habits were not analyzed temporally. The results of this study cannot be extrapolated to the general population of elderly people either, as geographical location influences dietary habits [23]; for example, only 17% of our sample adhered to the recommended vegetable intake, and this may not be the usual level of consumption in other places. Also, level of education is a socioeconomic factor that can moderate the relationship between vegetable intake and MetS [76], and in our sample, 40.5% had a high level of education (university level), a proportion higher than normal among older people. Therefore, it would be interesting to carry out studies in other geographical areas to uncover how vegetable consumption influences the development of MetS through longitudinal and cross-sectional research in a greater number of elderly people.

In this study, the potential influence of significant confounding variables associated with MetS, such as inadequate consumption of fruit and nuts, age, sex, and level of education, was taken into account to identify inadequate vegetable consumption as an independent factor for the presence of MetS. Therefore, another limitation arose in those important variables related to MetS, such as physical activity, socioeconomic status, total calorie intake, and risk habits (smoking, stress, and high alcohol consumption), which were not quantified in our study. Also, residual confounding from other dietary factors may have influenced the results.

## 5. Conclusions

The present study showed a beneficial association between an intake of at least two servings of vegetables per day and a lower prevalence of MetS. An important finding was that inadequate vegetable consumption was identified as an independent variable associated with the prevalence of MetS in older people, even when taking inadequate consumption of fruit and nuts and age, sex, and level of education into account. Promoting increased vegetable consumption through public health nutrition programs emerges as an appropriate strategy for enabling healthier aging in this population group with very low adherence to the recommended intake of vegetables and a high prevalence of MetS.

## Figures and Tables

**Table 1:** Socio-demographic characteristics.

	Total (*n* = 264)	
	N	%
Gender		
Men	103	39.0
Women	161	61.0
Age group		
65–69	88	33.3
70–74	98	37.1
75–79	78	29.6
Marital status		
Married/partnered	167	63.2
Separated	16	6.1
Widowed	48	18.2
Single	33	12.5
Level of education		
University	107	40.5
Secondary school	74	28.0
Primary school	76	28,8
Incomplete primary school	7	2.7

**Table 2:** Vegetable consumption according to gender.

	<2 Servings/Day (*n* = 219)	≥2 Servings/Day (*n* = 45)	*p*	Total (*n* = 264)
	*n* (%)	*n* (%)		*n* (%)
Men	92 (89.3)	11 (10.7)	0.028	103 (39.0)
Women	127 (78.9)	34 (21.1)		161 (61.0)

*p*: chi-square test.

**Table 3:** Diagnostic parameters related to metabolic syndrome.

	Total (*n* = 264)	%
Abdominal obesity	208	78.8
Diagnostic parameters for MetS		
Arterial hypertension	210	79.5
Hyperglycemia	84	31.8
Hypertriglyceridemia	59	22.3
Low HDL-c levels	52	19.7
Number of variables for a diagnosis of MetS		
None	38	14.4
1	102	38.6
2	73	27.6
3	40	15.2
4	11	4.2
Metabolic syndrome	106	40.2

**Table 4:** Univariate logistic regression analysis of the potential independent variables with metabolic syndrome.

Variables	OR	95% CI	*p*
Use olive oil for cooking	2.27	0.37–13.83	0.373
Oil (≥4 tablespoons/day)	1.01	0.61–1.67	0.967
Vegetables (≥2 servings/day)	2.37	1.14–4.92	0.021
Fruit (≥3 pieces/day)	1.35	0.82–2.23	0.237
Red meat, hamburgers, sausage (<1 serving/day)	1.05	0.39–2.84	0.929
Butter, margarine, cream (<1 serving/day)	1.04	0.52–2.08	0.904
Carbonated/sweetened beverage (<1 serving/day)	1.38	0.67–2.85	0.380
Wine (≥7 glasses/week)	1.02	0.60–1.73	0.941
Legumes (≥3 servings/week)	0.80	0.48–1.35	0.411
Fish/seafood (≥3 portions/week)	1.24	0.76–2.03	0.393
Commercial pastries (<2 servings/week)	1.01	0.62–1.66	0.962
Nuts (≥3 portions/week)	2.04	1.21–3.42	0.007
Prefers chicken, turkey, or rabbit instead beef, pork, hamburgers or sausages	1.20	0.73–1.98	0.471
Sofrito with cooked vegetables, pasta, or rice (≥2 times/week)	0.76	0.42–1.38	0.368
Sex (reference women)	1.66	1.00–2.74	0.049
Age (years)	1.01	0.96–1.08	0.623
Level of education			
University (reference)	0.86	0.46–1.58	0.615
Secondary school	1.14	0.63–2.07	0.662
Primary school	1.12	0.24–5.24	0.889
Incomplete primary school			

*p*: Wald test; OR: odds ratio; and CI: confidence interval.

**Table 5:** Multivariate regression logistic analysis of the M0 and M1 models.

		M0 *			M1 **	
Variables	OR	95% CI	*p*	OR	95% CI	*p*
Vegetables (≥2 servings/day)	1.96	0.98–4.17	0.082	2.21	1.06–4.63	0.035
Fruit (≥3 pieces/day)	1.20	0.71–2.02	0.490			
Nuts (≥3 servings/week)	1.93	1.13–3.28	0.016	1.95	1.15–3.29	0.013
Sex (reference women)	1.56	0.91–2.67	0.108			
Age (years)	0.98	0.92–1.04	0.442			
Level of education						
University (reference)						
Secondary school	0.86	0.45–1.64	0.654			
Primary school	1.17	0.62–2.22	0.629			
Incomplete primary school	1.43	0.28–7.14	0.667			

*p:* Wald test; OR: odds ratio; and CI: confidence interval. The area under the ROC curve (AUC): * M0: 0.641; ** M1: 0.61.

## Data Availability

The data presented in this study are available upon request from the corresponding author. The data are not publicly available due to privacy protection.

## Supplementary Materials

The following supporting information can be downloaded at https://www.mdpi.com/article/10.3390/nu16234101/s1, Table S1: Types of consumption for the variables in the MEDAS-14 questionnaire.
