# Supplementary material for: Daily Intake of Two or More Servings of Vegetables Is Associated with a Lower Prevalence of Metabolic Syndrome in Older People
Source: Nutrients. 2024 Nov 28;16(23):4101. doi: 10.3390/nu16234101 (PMC11643602; doi:10.3390/nu16234101)
Supplement: Supplementary file 1 [file nutrients-16-04101-s001.zip › nutrients-3278161-supplementary.pdf]

Table S1. Types of consumption for the variables in the MEDAS-14 questionnaire

Variables	Recommended	Inadequate
**Olive oil for cooking**	yes	No
**Oil (tablespoons/day)**	≥ 4	< 4
**Vegetables (servings (200g)/day)**	≥ 2	< 2
**Fruit (pieces/day)**	≥ 3	<3
**Red meat, hamburgers, sausage (servings (100-150g)/day)**	< 1	≥ 1
**Butter, margarine, cream (serving (12g)/day)**	< 1	≥ 1
**Carbonated/sweetened beverage (servings/day)**	< 1	≥ 1
**Wine (glasses (100 mL)/week)**	≥ 7	< 7
**Legumes (servings (150 g)/week)**	≥ 3	<3
**Fish (100-150g) /seafood (200g) (portins/week)**	≥ 3	<3
**Commercial pastries (servings/week)**	<2	≥ 2
**Nuts (portions (30g)/week)**	≥ 3	< 3
**Prefers beef, pork, hamburgers or sausage instead chicken, turkey or rabbit**	No	Yes
**Sofrito with cooked vegetables, pasta or rice (times/week)**	≥ 2	< 2
